# Cross-species comparison of AlphaFold-derived G protein-coupled receptor structures reveals novel melatonin-related receptor in *Neurospora crassa*

**DOI:** 10.1371/journal.pone.0318362

**Published:** 2025-01-28

**Authors:** Cathryn S. D. Maienza, Guillaume Lamoureux, Kwangwon Lee

**Affiliations:** 1 Center for Computation and Integrative Biology, Rutgers, The State of New Jersey, Camden, NJ, United States of America; 2 Department of Chemistry, Rutgers, The State University of New Jersey, Camden, NJ, United States of America; 3 Department of Biology, Rutgers, The State University of New Jersey, Camden, NJ, United States of America; Karlsruhe Institute of Technology: Karlsruher Institut fur Technologie, GERMANY

## Abstract

Melatonin, a molecule with diverse biological functions, is ubiquitously present in living organisms. There is significant interest in understanding melatonin signal transduction pathways in humans, particularly due to its critical role in regulating the sleep-wake cycle. However, a knowledge gap remains in fully elucidating the mechanisms by which melatonin influences circadian regulation. To bridge this gap, there is a growing need for a model system to study the role of melatonin in circadian clocks, with *Neurospora crassa* being a promising candidate. As a first step in this investigation, we focused on identifying melatonin receptors in *N*. *crassa*. Given the lack of sequence similarity between potential receptors in this fungus and known human melatonin receptors, we utilized structural similarity analysis through AlphaFold2. This approach led to the identification of a strong candidate gene, *gpr-3*, which shares structural similarities with human melatonin receptors. Experimental validation confirmed that the removal of GPR-3 from cells results in the absence of melatonin signaling. This proof-of-concept study underscores the potential of *N*. *crassa* as a model organism for circadian research and demonstrates the broader applicability of using AlphaFold2, especially when sequence similarity does not lead to candidate genes, for identifying novel receptors across different species.

## Introduction

Circadian rhythms, or biological processes that follow an endogenous 24-hour cycle, are found across all kingdoms of life, allowing organisms to adapt to daily fluctuations in external stimuli. Time-of-day cues called “zeitgebers” cause expressional and posttranslational alterations to genes comprising an organism’s molecular clock, thus entraining, or syncing, the clock to external conditions [1–3]. Although the genes involved differ on a species level, the general mechanistic composition of the molecular clock, or circadian oscillator, is conserved across vertebrates, invertebrates, and fungi: a positive element promotes the expression of a negative element, which accumulates and undergoes posttranslational modifications eventually forming a complex which represses expression of the negative element. The cycle starts anew when the negative element reaches a specific level of phosphorylation and/or is degraded, and expressional repression is relieved [4–7]. This conserved transcriptional-translational feedback oscillator (TTFO) allows for experimental comparisons between species of molecular responses to zeitgebers, as has been done for decades in the field of chronobiology using the filamentous fungi *Neurospora crassa* and mammalian systems.

Although TTFO entrainment to classic zeitgebers like light and temperature have been widely studied and are well categorized in model organisms across kingdoms [8–11], the same cannot be said of the hormonal zeitgeber known as melatonin. Melatonin is a widely conserved molecule derived from the tryptophan/serotonin biosynthesis pathway and enzymes which catalyze its biosynthesis like serotonin N-acetyltransferase (AANAT) and N-acetylserotonin O-methyltransferase (ASMT) have been found in animals, plants, fungi, and bacteria [12,13]. Most of what is known about melatonin has come from vertebrate systems, focusing on its pharmacological properties in humans. Melatonin has therapeutic effects for a wide range of physiological processes including detoxification of free radicals, cardiovascular health, fetal development, and tumor suppression [14,15]. The hormone is known for its role in sleep regulation, acting as an endogenous zeitgeber synthesized and secreted into the circulatory system from the pineal gland during subjective night [15,16]. Secretion of melatonin in animals adheres to a circadian rhythm: levels of melatonin detectable through blood and saliva begin to rise at the onset of subjective night, peaking at midnight before gradually returning to basal levels early in the subjective morning [17]. Despite a sparsity of research done in Neurospora regarding melatonin, the hormone was found to follow a similar secretion pattern into surrounding liquid media in cycling and constant photoperiodic conditions [12].

In mammalian systems, melatonin has been found to alter the amplitude and rhythmicity of gene expression within the TTFO. The positive elements *Bmal1* and *Clock (Clk)* and negative elements *Per* and *Cry* appear to have tissue-specific expression responses to melatonin treatment, generally manifesting as reduced expression of one gene, usually *Per*, or combinations of them [15,18]. It is currently believed the observed changes in the TTFO in response to melatonin are due to suppression of the highly conserved cAMP/PKA signaling pathway and of cAMP-responsive element (CRE)-binding protein (CREB), a known regulatory element of *Per*, recruiting the positive elements BMAL1 and CLK consequently promoting *Per* expression [19,20]. Although nothing is known of melatonin signaling in Neurospora, PKA is known to post-translationally-modify the positive element White Collar 1 (WC-1) leading to destabilization of the protein and expressional inhibition of the negative element *frequency* (*frq*). PKA is also able to phosphorylate FRQ protein, in this case stabilizing the core clock element and slowing its degradation [21,22]. Given the existence of a conserved cAMP-dependent kinase able to alter expression of core clock elements of the TTFO in both mammals and Neurospora, a conserved mechanism for melatonin signaling is possible.

Decreased cellular cAMP concentration in response to melatonin is attributed to the activity of two Class A/Rhodopsin Family G protein-coupled (GPCR) melatonin receptors, MT1 and MT2, whose G**α**i subunits inhibit the activity of adenylyl cyclase (AC) and production of cAMP [23]. In recent years, much research has been done on the protein-protein interactions between MT1 and MT2, as the receptors are often found as hetero- or homodimers with themselves, or other melatonin-related GPCRs like GPR50 that otherwise do not bind directly with the hormone [24]. Understanding how MT1 and MT2 interact is a crucial advancement for pharmacology, given that the receptors have different downstream signaling pathways depending on their dimerization and tissue of origin [23]. Finding one, potentially multiple, melatonin receptors in a genetically simple, yet orthologous, eukaryotic model system like Neurospora would increase the efficiency by which these hetero-/homodimer dynamics are studied experimentally.

Melatonin receptors have been found in complex eukaryotic organisms such as nonmammalian vertebrates, invertebrates, and plants [13]. However, attempting to identify orthologous receptors between kingdoms through primary protein sequence proves challenging. While vertebrate melatonin receptors are similar enough to appear with high percentage identity in a BLASTP search, known plant and invertebrate receptors align poorly with MT1 and MT2. When compared to the plant melatonin receptor PMTR-1 found in *Arabidopsis thaliana* (AN: Q94AH1.1), human MT1 (AN: NP_005949.1) aligns with only 21% coverage and roughly 32% percent identity [25]. The sequence for AccMTNR1A (AN: ASN64549.1), a melatonin receptor found in the honeybee species *Apis cerana cerana*, is too dissimilar in sequence to align with MT1 [26]. Interestingly, despite low sequence homology, nearly all melatonin-binding receptors are transmembrane GPCRs, suggesting conservation of structure and function rather than primary amino acid sequence. Given the widespread conservation of melatonin, it is possible that the primary sequence of MT1 and orthologous proteins were allowed to diverge during evolution, so long as the ligand-binding pocket remained compatible with melatonin. Alternatively, the lack of sequence homology in melatonin receptors could be the result of convergent evolution, such that a receptor for melatonin arose independently in different kingdoms. For this reason, we reasoned that structural comparisons of MT1 to GPCRs with unknown functions could prove invaluable for identifying novel melatonin receptors across kingdoms.

In this study, we aim to identify a novel melatonin receptor in *N*. *crassa* by comparing structures of human of MT1 and MT2 with the 43 known fungal GPCRs, some of which have unknown functions [27]. Traditionally costly and time-consuming structural protein analyses have been made more accessible by AI-based programs such as Google DeepMind’s AlphaFold [28] used in combination with publicly available alignment tools like Dali protein alignment [29] and the more recent Foldseek [30]. Through this methodology, we identified a group of fungal GPCRs that form a sister clade with MT1 and MT2 based on their structural similarity, focusing on a functionally uncharacterized fungal receptor, *gpr-3*. We found that genetic knockouts of *gpr-3* are insensitive to treatment with melatonin when compared to the wild type, supporting our hypothesis that *gpr-3* is a novel melatonin-related receptor in fungi. This study represents the first identification of a melatonin-related receptor in fungi as far as the authors’ knowledge and proposes AlphaFold2/Dali structural comparisons as a powerful tool for hypothesis generation when attempting to identify orthologous proteins in other model organisms.

## Results

### Alphafold2/Dali structural alignment for human GPCRs recapitulates class relationships

**Fig 1:**
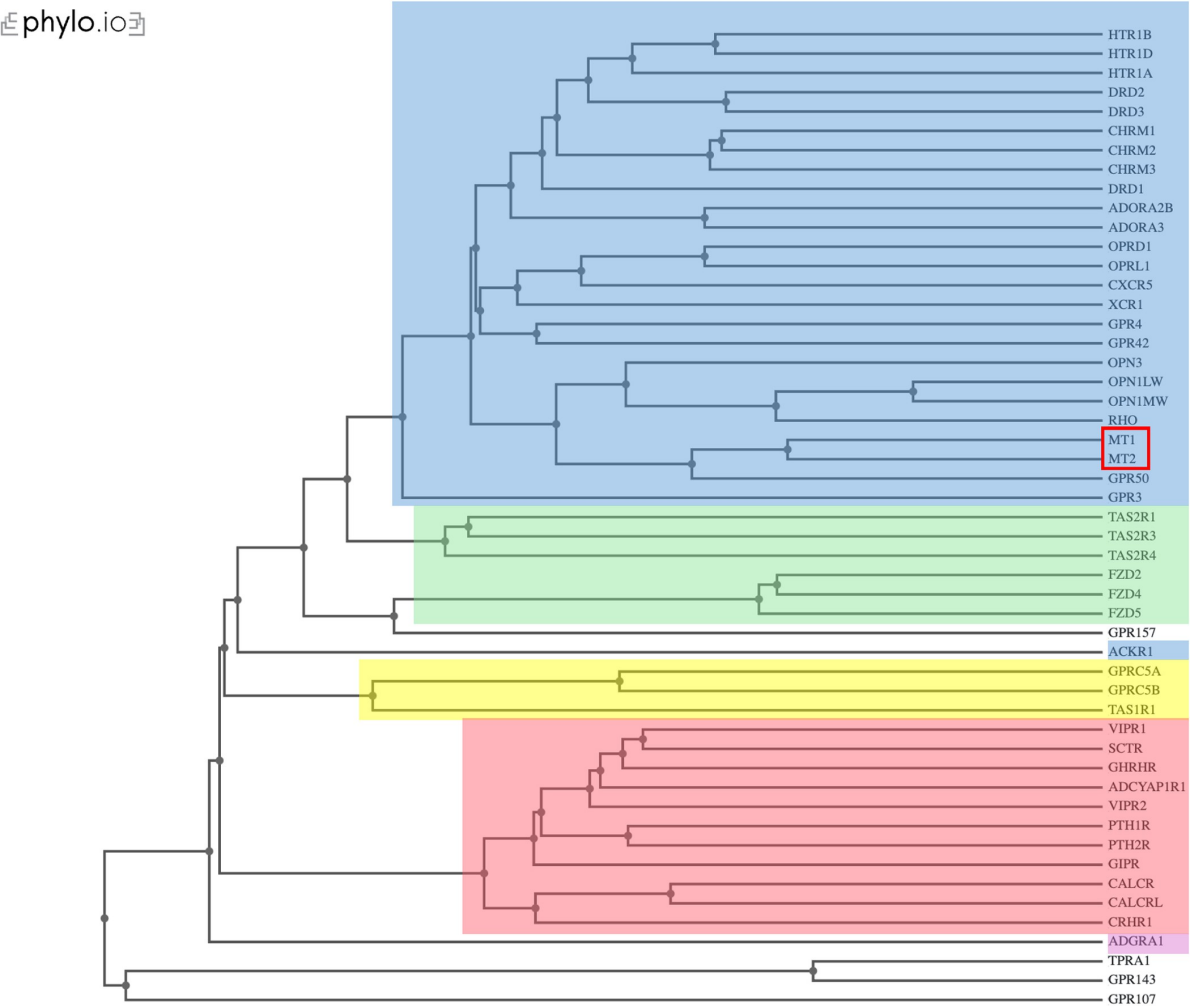
Dendrogram of structural relatedness for 51 human GPCRs including MT1/MT2. FASTA protein sequences for 51 randomly sampled GPCRs were structurally predicted using AlphaFold2 and compared using Dali All Against All. MT1/MT2 outlined in red. Color boxes denote GPCR class: Class A/Rhodopsin (blue), Class B/Secretin (red), Class C/Glutamate (yellow), Class F/Frizzled/Taste (green), Class *Adhesion* (purple), other (no color). Dendrogram formatted using Phylo.io.

As proof of concept and to test AlphaFold2’s accuracy with GPCR structural predictions, 51 human GPCRs from each of the five recognized GPCR classes were randomly sampled relative to class size (Methods). Each of the five classes are established based off structural similarities and ligand-binding capabilities possessed by its protein family, therefore providing a good test sample for AlphaFold2’s ability to predict functionally relevant structures [31,32]. The AlphaFold-predicted structures of the sample set were aligned using Dali All Against All to visualize their structural relatedness (Fig 1). With few exceptions, the GPCR’s were grouped together into clades with their respective family members, in many cases recapitulating the specific phylogenetic relationships determined through sequence analysis [31]. Similarities to previously determined phylogenies often have minor inconsistencies, such as the individual relationships for HTR1B/D/A and DRD1/2/3 in Class A matching the sequence phylogeny but disagreeing with each group’s proposed relationship to CHRM1/2/3. Nonetheless, the relationships of GPCRs most closely related to MT1 and MT2 as determined by structure, such as GPR50, OPN3, RHO, and OPN1LW/MW, were identical to those proposed by sequence alignment. Interestingly, GPR50 which itself cannot bind to melatonin yet interacts with MT1 forms a sister clade with MT1 and MT2, hinting at their related function albeit different ligand binding capabilities. The resulting similarities between previous literature and our AlphaFold2-derived structural phylogeny provided a sound framework for which to continue structural comparisons of MT1 and MT2 with fungal GPCRs.

### Alphafold2/Dali structural comparison reveals fungal sister clade to MT1/MT2

**Fig 2:**
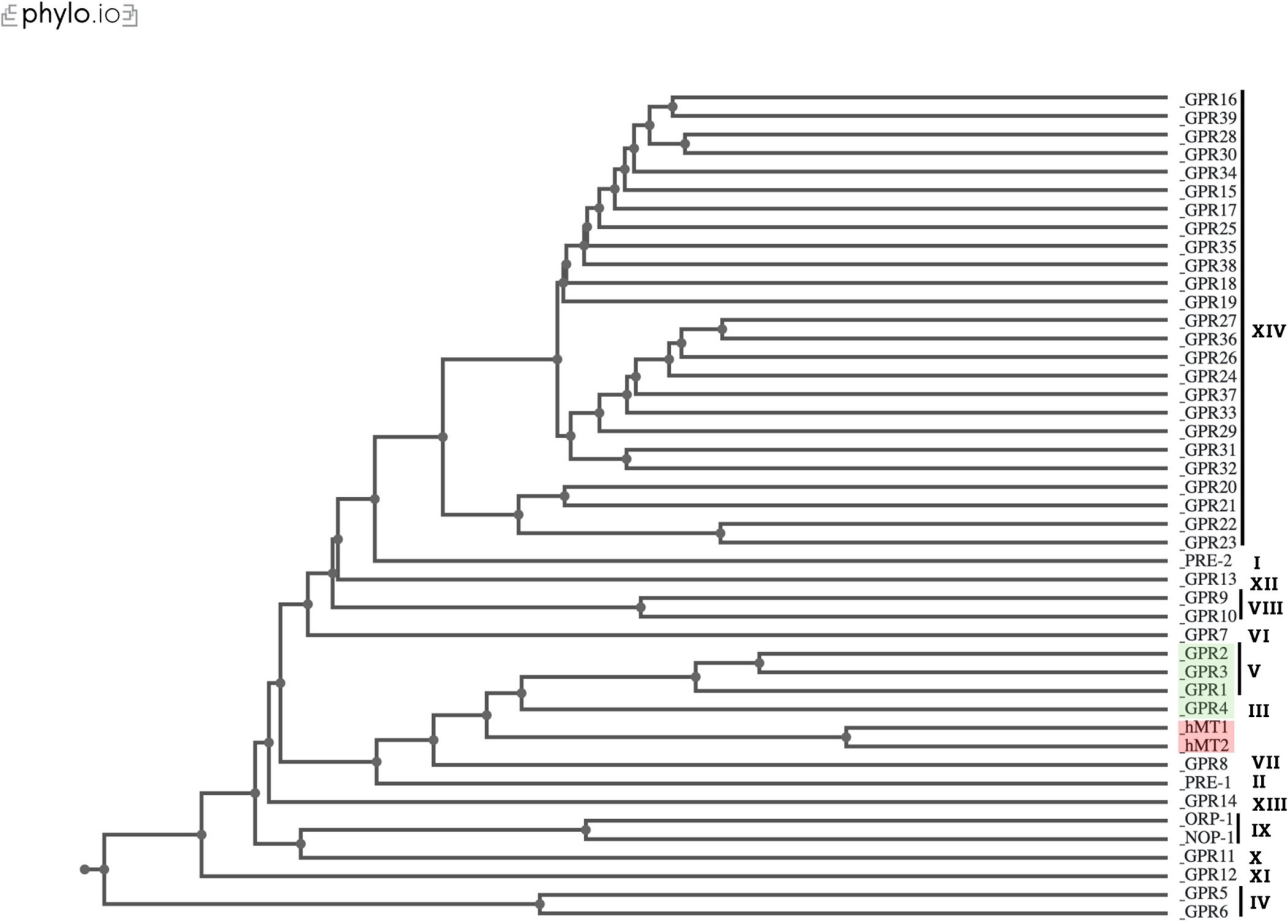
Dendrogram of structural relatedness between 43 fungal GPCRs and MT1/MT2. FASTA protein sequences for the 43 identified fungal GPCRs and human MT1/MT2 were structurally predicted using AlphaFold2 and compared using Dali All Against All. A sister clade is formed with four fungal GPCRs, GPR-1-4, marked with a green box. The clade containing MT1/MT2 is shown with a red box. Roman numerals indicate which of the 14 fungal GPCR class each GPCR falls into as outlined in Cabrera et al., 2015. Dendrogram formatted using Phylo.io.

To identify fungal GPCRs structurally like human MT1/MT2, structural predictions were generated with AlphaFold2 for the 43 GPCRs in *N*. *crassa* (detailed in Cecon et al., 2018) and MT1/MT2. The proteins were structurally aligned using Dali All Against All to produce a dendrogram (Fig 2). A group of four fungal GPCRs, GPR-1-4, were most closely related to the structures of MT1/MT2 forming a sister clade to the human receptors. GPR-1-3 are members of the “cAMP receptor-like” Class V GPCR family while GPR-4 is a “carbon sensory” Class III GPCR in *N*. *crassa*; the grouping of these GPCRs in relation to GPCR Class is recapitulated in the dendrogram, as GPR-1-3 are more alike than any are to GPR-4. The functions of GPR-1 and GPR-4 have been experimentally confirmed: *gpr-1* mutants exhibit abnormal female sexual reproductive organ development and *gpr-4* mutants struggle to adapt to different carbon sources [33,34]. The remaining two proteins, GPR-2 and GPR-3, have no known functions and have relatively similar similarity scores to MT1 (Z-score and %ID for GPR-2: 23.3 and 12%, for GPR-3: 21.1 and 14%). We chose to focus on one receptor, GPR-3, for further experimental validation of melatonin sensitivity. Dali pairwise alignments of GPR-3 to MT1/MT2 highlight structural equivalency of amino acid positions in GPR-3 relevant to the ligand-binding pocket in MT1/MT2, such as the YPYP motif (Y79-P82) and C177/F179 found within extracellular loop 2 (ECL2) (S2 Fig) [35].

### Knockouts of *gpr-3* lack FRQ phase shift in response to melatonin

**Fig 3:**
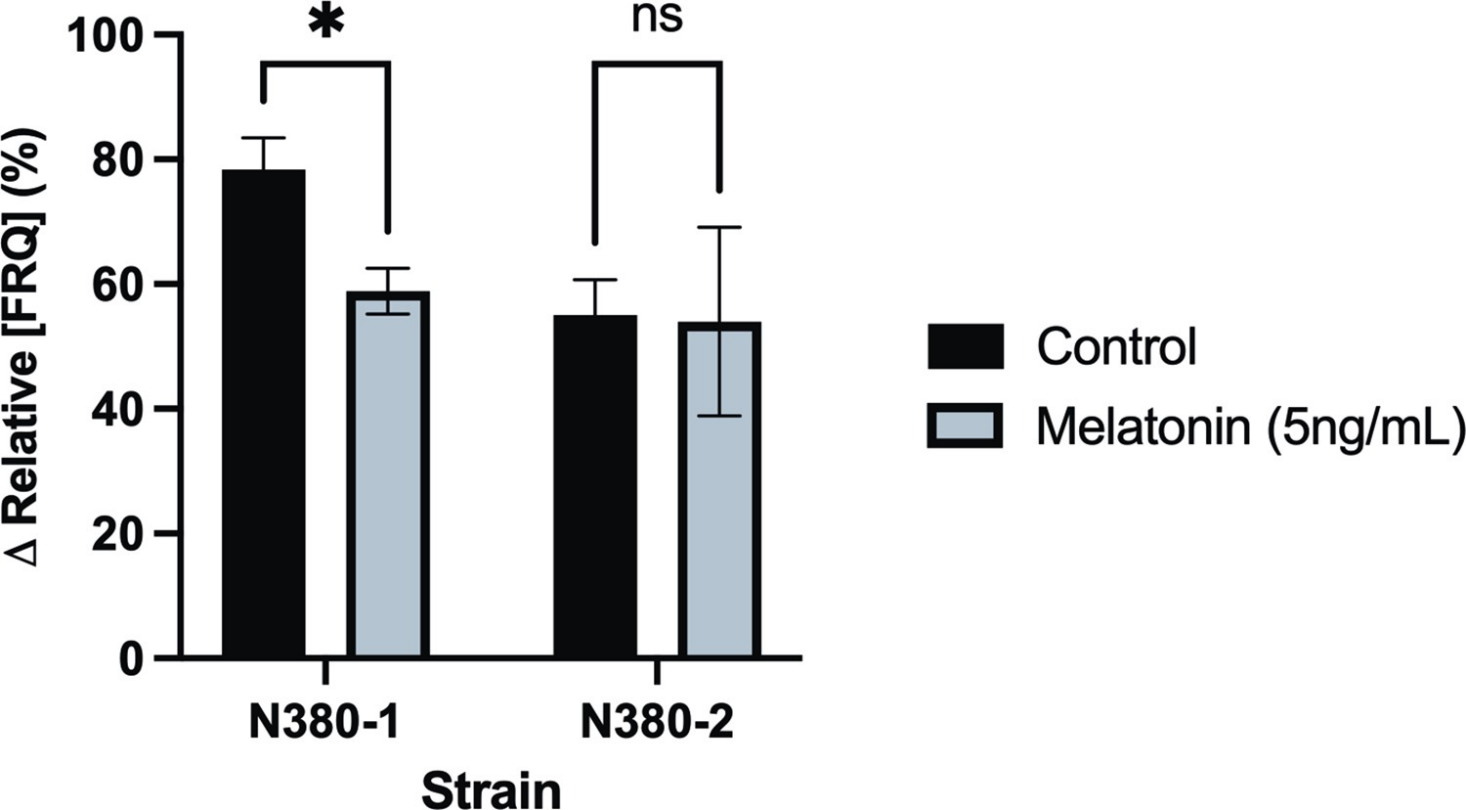
Genetic knockouts of *gpr-3* are insensitive to melatonin-induced phase shifts of TTFO negative element FRQ. Difference in FRQ::LUC between *gpr-3*^*-*^ (N380-2, n = 3) and *gpr-3*^*+*^ (N380-1, n = 3) strains of *Neurospora crassa* two hours after treatment with 5ng/mL melatonin in 1x fructose/glucose/sorbose media (FGS). Control group was treated with sterile water. Two-way ANOVA revealed significant effect of melatonin treatment on N380-1 (p = 0.02) but not in the strain lacking *gpr-3* (p = 0.07). FRQ::LUC–luciferase translational reporter for key clock component Frequency, *–p<0.05, ns–not significant.

While our AlphaFold2/Dali approach provided us with the candidate gene *gpr-3*, experimental validations of melatonin-insensitivity phenotypes in Neurospora strains lacking *gpr-3* were conducted to test our hypothesis of GPR-3 as a fungal melatonin-related receptor. In mammalian systems, melatonin affects the relative abundance of key clock elements downstream of the melatonin signaling pathway [19,20]. To test this in Neurospora, we used a luciferase translational reporter of the negative clock element FRQ (FRQ::LUC) to measure changes in FRQ oscillation rates following treatment with 5 ng/mL melatonin in constant dark conditions (Fig 3).

FRQ::LUC levels provide a reliable proxy for circadian time (CT), where CT0/24 corresponds to the minimum FRQ level at the start of the subjective day, and CT12 represents the maximum level at the start of the subjective night. This allows visualization of phase shifts or resetting of the circadian TTFO by external stimuli, also known as zeitgebers, such as melatonin [36,37].

Under constant dark conditions, FRQ levels naturally decline at CT18, serving as a baseline. This expected oscillation pattern was observed in both wild-type (N380-1) and *gpr-3* knockout (N380-2) strains; however, the rate of decline was less robust in N380-2. Following melatonin treatment, the wild-type strain N380-1 exhibited a significant reduction in the rate of FRQ oscillation (two-way ANOVA, p = 0.02), supporting the hypothesis that melatonin influences TTFO elements in Neurospora. This response was absent in the knockout strain N380-2, as the rate of FRQ decline did not significantly differ after melatonin treatment. These findings suggest that GPR-3 plays an essential role in mediating melatonin signaling pathways in Neurospora.

### Knockouts of *gpr-3* exhibit reduced sensitivity to melatonin-induced cAMP fluctuations

**Fig 4:**
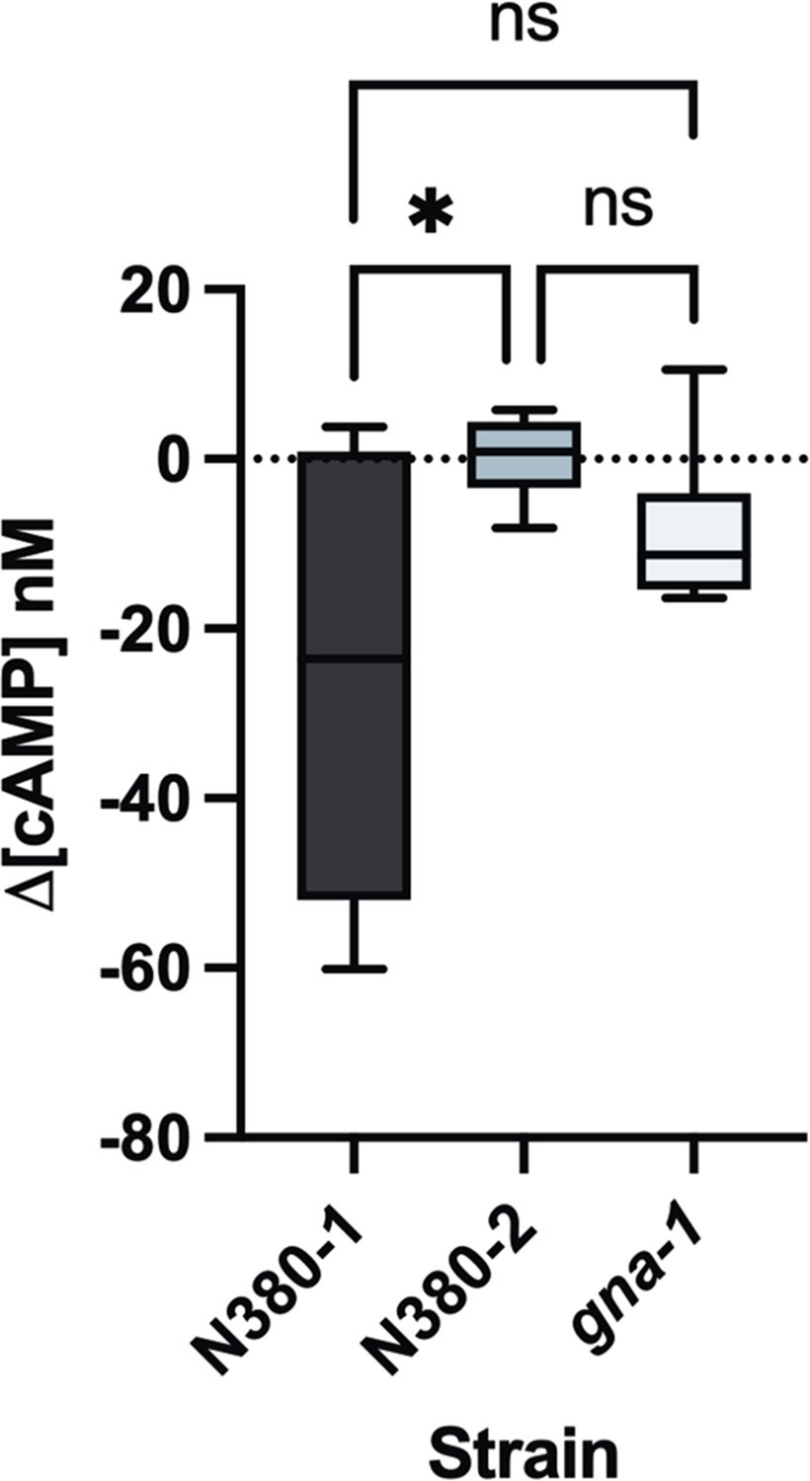
Knockouts of *gpr-3* lack cAMP signaling response to melatonin. Difference in relative cAMP concentration between *gpr-3*^*+*^ (N380-1, n = 6), *gpr-3*^*-* (^N380-2, n = 6), and *gna-1*^*-*^(n = 6) strains after 10-minute treatment with 5ng/mL melatonin in sterile water. Untreated group was treated only with water. One-way ANOVA revealed statistically significant decrease of cAMP in response to melatonin treatment in N380-1 when compared to N380-2 (p = 0.036). There was no significant difference between N380-1 or N380-2 with *gna-1* (p = 0.204, p = 0.607, respectfully. nd–no difference, *–p<0.05.

The binding of melatonin to MT1 or MT2 results in an overall decrease in cAMP concentration in mammalian cells [20,23]. In a preliminary study examining the effects of a range of melatonin concentrations on cAMP levels in our wild type, we observed robust declines in cellular cAMP levels from concentrations as high as 50μg/mL to the physiologically relevant range of 5ng/mL of melatonin (S1 Fig). Therefore, to test *gpr-3* as a melatonin-related receptor we measured changes in cAMP levels after treatment with physiologically relevant concentrations of melatonin in a *gpr-3*^*-*^ knockout (N380-2), its wild type sister strain (N380-1), and a knockout of the G**α**i subunit *gna-1*. In mammalian melatonin receptors the G**α**i subunit of MT1/MT2 inhibits cAMP production [23], leading us to hypothesize a conserved G-protein preference for receptor-mediated melatonin signaling in Neurospora. Previous research has found that one of the three fungal G**α**i subunits, *gna-1*, acts downstream of *gpr-1* and *gpr-4* [33,34], thus we used *gna-1* as a negative control. Samples of the three strains were entrained to cycling conditions (LD 12:12) before treatment at zeitgeber time 18 (ZT18) with a 5ng/mL melatonin solution for 10 minutes and assayed for changes in relative cAMP levels (Fig 4). After treatment with melatonin, the *gpr*^*+*^ strain N380-1 experienced a decrease in cellular cAMP averaging around a 26 nM, with a maximum observed decrease of up to 60 nM. The decrease in cAMP concentration seen in N380-1 is consistent with what is known about human MT1 and MT2, suggesting a conserved signaling molecule pathway. The *gpr-3*^*-*^ strain N380-2 showed an average increase in cAMP levels of approximately 0.25nM while *gna-1* exhibited an average decrease of 8.74nM, compared to the control strain N380-1. Interestingly, fluctuations in cellular cAMP levels were only significantly different between the sister strains N380-1 and N380-2 (one-way ANOVA, p = 0.036). Despite the cAMP assay results from *gna-1* lacking a statistical difference from those of N380-1 and N380-2, melatonin-induced alterations in cellular concentration of cAMP were clearly observed in the wild type yet not in strains lacking *gpr-3*, and to a lesser extent in *gna-1*. These findings support our hypothesis that *gpr-3* is involved in the melatonin signaling response in Neurospora and the cAMP signaling pathway is a conserved downstream mechanism of receptor-mediated responses to melatonin.

## Discussion

Through the combined application of AlphaFold2 and Dali, we conducted a cross-species structural comparison of human melatonin receptors MT1 and MT2 to all 43 fungal GPCRs identified in *N*. *crassa*, hypothesizing that a novel fungal melatonin receptor would have a conserved tertiary structure to those found in mammals. We identified a group of four fungal receptors, GPR-1-4, which formed a sister clade with MT1/MT2 and used further experimental tests to validate one protein, GPR-3, as a candidate melatonin-related receptor in Neurospora. Knockout strains of *gpr-3* exhibited an insensitivity to melatonin-induced phase shifts in the natural oscillation of FRQ, the negative regulatory element of the Neurospora TTFO. By assaying cAMP levels after treatment with melatonin, we observed significant reductions in cellular cAMP concentrations in our wild type but not in strains lacking *gpr-3*. Collectively our findings support our hypothesis that Neurospora possess a melatonin-sensitive GPCR structurally similar to MT1/MT2 and present Neurospora as a useful model for future research into the genetic conservation of the melatonin signaling pathway across eukaryotes.

Structural and comparative genomics are valuable tools for a wide variety of biological fields, particularly in understanding disease evolution and progressing advancements in pharmacology [38,39]. With the advent of AlphaFold and its ability to predict protein structures with high efficiency and accuracy, combining structural and comparative genomics such as we did through our use of two free-to-use programs, AlphaFold2 and Dali, is more accessible than ever. Our work shows the power of this methodology to identify potential orthologs to human melatonin receptors in *N*. *crassa*, thus providing a simple eukaryotic model organism for melatonin pharmacology. Although the identification of a fungal melatonin receptor was the focal point of our study, the combined application of AlphaFold2/Dali could be further utilized to research GPCRs in humans and Neurospora. For example, there remain “orphan” GPCRs with unknown ligands in many eukaryotic organisms like mammals and fungi, whose functions could be hypothesized through a similar methodology to ours [40]. Our approach also provides insights for molecular evolution: cross-referencing previously determined GPCR class relationships in humans based on sequence alignments [31] with phylogenies derived from AlphaFold2/Dali adds an additional layer of understanding to the evolution of GPCRs in eukaryotes. Our unique approach to structural and comparative genomics is applicable to many biological questions and when used in conjunction with experimental validation is a powerful tool for hypothesis generation.

This study was validated experimentally through FRQ::LUC and cAMP level assays in *gpr-3*^*-*^ strains, providing the first evidence of a novel melatonin-related receptor in *N*. *crassa*. Given the seemingly conserved, circadian secretion of humoral melatonin between *N*. *crassa* and humans [12,17], we hypothesize that much of the molecular mechanisms of melatonin are conserved between them, and potentially all eukaryotes. Many questions do remain regarding the entire Neurospora TTFO response to melatonin and the specifics of the *gpr-3*-mediated melatonin signaling pathway. We currently suspect PKA as a potential bridge between the cAMP signaling pathway and the TTFO in Neurospora, as has been proposed in mammals [19,20]. We witnessed a significant decrease in the natural decline of FRQ protein in response to melatonin. In mammals, it has been observed that expression of the negative element *Per* and sometimes *Cry* decreases after treatment with melatonin [15], potentially hinting at a conserved mechanism of modulating a negative TTFO element. Future examinations of the relative transcript and protein levels of key clock elements WC-1, WC-2, and FRQ in response to melatonin would provide valuable insights into the receptor-mediated zeitgeber response to the hormone in Neurospora.

Regarding a conserved cAMP signaling response downstream of GPR-3, our wild type exhibited a significant decrease in cellular cAMP after melatonin treatment that was not observed in *gpr-3* knockouts, supporting our hypothesis that GPR-3 is a melatonin-related receptor in Neurospora. Interestingly, our negative control, *gna-1*, was not significantly different from the wild type or knockout though still experiencing a markedly reduced response to melatonin. Given that there are three G**α**i genes in Neurospora, it is possible that *gna-2* or *gna-3* are the preferential G-protein for GPR-3 [41,42]. However, this result could also highlight a difference in the pharmacological profile of GPR-3 to MT1/MT2. It is widely observed that the preferred G-protein subunit for both human melatonin receptors is G**α**i, though MT1 has been found to couple with G_q/11_ and G_s_ subunits [43,44]. While our findings revealed a shared response to melatonin between GPR-3 and MT1/MT2, namely a marked decrease in cellular cAMP levels, more research is needed to fully characterize GPR-3 as an authentic MT1/MT2 ortholog. Further research into the preferred subunit of GPR-3, as well as an examination of cAMP synthesis and signaling in response to melatonin through GPR-3 will add to our comprehension of GPR-3’s species-specific pharmacological behavior.

In the current study, we presented a single candidate melatonin-related receptor in Neurospora, namely GPR-3. However, a question we are working on is whether Neurospora possess multiple melatonin receptors, as is observed in vertebrates. We did not study GPR-2, the other fungal GPCR with unknown functions, yet it remains a protein of interest given its structural similarity to MT1/MT2. We also do not rule out the possibility of multiple functions in GPR-1 and GPR-4 and are currently working to characterize the melatonin response in single and multi-knockouts of each gene. For example, though previous studies have not found shared functions between these proteins, it has been proposed in the case of GPR-1, a protein required for proper female reproductive organ development, that G-protein-independent functions may exist [33]. It is possible that like human GPR50, one of the other three fungal GPCRs could lack the ability to bind to melatonin but interact with and inhibit an authentic melatonin receptor [23]. Given that the multiple melatonin receptors in mammals can interact with one another and experience differential preferences in G-protein subunit binding, there are many useful applications for a simple model organism with orthologous proteins to MT1, MT2 and GPR50. Identifying multiple melatonin-related receptors in both mammals and fungi would also support the idea that other eukaryotes such as plants and insects also possess yet to be identified receptors that could be found through a similar methodology to our own.

By adopting a structural and comparative approach through the combined use of AlphaFold2 and Dali, we identified a GPCR in *N*. *crassa*, GPR-3, that is structurally like human MT1 and MT2. Experimental validation of sensitivity to melatonin in knockouts of *gpr-3* show reduced downstream responses to the hormone in FRQ oscillation rates and intracellular cAMP levels, supporting our hypothesis of GPR-3 as the first melatonin-related receptor to be identified in *N*. *crassa*. Our innovative approach to finding orthologous proteins across kingdoms presents an easily accessible methodology that could hasten the identification of pharmacologically relevant proteins in other model organisms.

## Materials and methods

### Structural comparison of pharmacologically relevant human GPCRs using AlphaFold2/Dali

A list of 500 human GPCRs were obtained from the IUPHAR/BPS Guide to Pharmacology database and randomly sampled, with specific inclusion of MT1 and MT2, relative to Class size [45]. 51 human GPCR’s were sampled in total from the 6 classes: 24 from Class A/Rhodopsin Family, 11 from Class B/Secretin Family, 1 from Class B/Adhesion Family, 3 from Class C/Glutamate Family, 6 from Class F/Frizzled Family, and 4 categorized as Other TM7 proteins. Class B/Adhesion and Class C/Glutamate had disparate sampling size due larger size (1,500+ aa) of GPCR’s in these classes; large proteins have low confidence scores in AlphaFold. FASTA files for the individual protein sequences were compiled and run through AlphaFold2 ColabFold BATCH with standard settings [46]. The models with the highest confidence (pLDDT score closest to 100) were then structurally aligned using Dali all against all comparison [29]. The dendrogram produced by Dali was saved as a phyloxml file and formatted using Phylo.io.

### Structural comparison of fungal GPCRs to MT1/MT2 using AlphaFold2/Dali

FASTA files with protein sequences for the 43 fungal GPCRs were obtained through FungiDB based off provided accession numbers [27]. Protein sequences for MT1 and MT2 were obtained through NCBI (MT1: NP_005949.1, MT2: NP_005950.1). All 45 FASTA files were input into ColabFold BATCH with standard settings [46]. For each protein, the output pdb file with the highest confidence score (pLDDT) was then input into the Dali All Against All alignment program. The dendrogram produced by Dali was saved as a phyloxml file and formatted using Phylo.io. Acquisition of individual similarity values for GPR-2 and GPR-3 against MT1 was done through Dali’s pairwise structure comparison.

### *N*. *crassa* strains used

A knockout of *gpr-3* (NCU09427) was obtained from the Fungal Genetics Stock Center (FGSC) Department of Microbiology, University of Kansas Medical Center, Kansas City Kansas. This strain was crossed with the FRQ::LUC^*bd-*^ bearing strain L4 (X716-4A) to create a *gpr-3*^*-*^*;FRQ*::*LUC*^*bd-*^ strain referred to as N380. X716-4A was a gift from Dr. Luis Larrondo at Pontificia Universidad Católica de Chile. From this cross, a “wild type” *gpr-3*^*+*^*;FRQ*::*LUC*^*bd-*^ strain and its knockout (*gpr-3*^*-*^*;FRQ*::*LUC*^*bd-*^) sister strain (N380-1 and N380-2, respectively) were used for the experimental portions of this study. For the cAMP assays, a knockout of the fungal G**α**i, *gna-1* (NCU06493) from FGSC was used as a negative control.

### Growth and circadian entrainment of samples

For use in the cAMP assay, N380-1, N380-2 and *gna-1* were cultured in 200ml flasks containing agar complete medium. The samples were incubated at 30°C for 24 hours and then transferred to a growing chamber set to 25°C with equinox photoperiodic conditions (LD 12:12). Samples were allowed to entrain for 5 days before being removed for preparation at zeitgeber time (ZT) 12. Samples of N380-1 and N380-2 used for the melatonin-induced phase shift luciferase assay experiment were cultured on minimal media slants for five days in LD 12:12 at 25°C.

### Preparations of melatonin solution and cAMP-Glo^TM^ reagents

Physiologically relevant ranges of melatonin found in liquid cultures of *N*. *crassa* are between 1-3ng/mL [12]. For this study, a melatonin solution of 5ng/ml in sterile water was prepared via serial 10-fold dilutions from a 1mg/20mL stock solution (prepared with 100% ethanol). Samples were prepared the day of the experiment and the same sterile water for the solution was used as a control. All reagents for the cAMP-Glo^TM^ kit were prepared and stored in accordance with the provided protocol for the 96-well format.

### FRQ::LUC microplate assay for melatonin-induced phase shifts

Five-day-old cultures of N380-1 and N380-2 were used to make 1mL conidia suspensions in sterile water. 125μL of each conidia suspension was added to 5mL 1x sorbose/glucose/fructose liquid media (1% sorbose, 0.05% glucose/fructose, FGS) along with 200μL of 1000X biotin solution and 200μL of 10mM D-luciferin (dissolved in sterile water). The plating mixtures were vortexted and 200μL of each suspension were aliquoted into 12 wells. A sterile microplate film was applied to minimize evaporation, and a sterile needle was used to create small holes for gas exchange over each well. The microplate was put into LD12:12 conditions at 25°C for one day to colonize before being put into a luminometer in constant darkness at 25°C. The luminometer firefly luciferase program was set to cycle a max length kinetic read twice (100 hours single, 200 hours total) at one-hour intervals. All strains contained a translational reporter of the negative element Frequency (FRQ) in the form of a FRQ::Luciferase (FRQ::LUC) fusion protein [47]. The levels of FRQ were monitored to observe when amounts of FRQ reached their peak (~CT12). Six hours after this peak (~CT18) the program was interrupted under red light and 3 wells from each strain were spiked with either 10μL of 5ng/mL melatonin in sterile water for the test group, or 10μL of sterile water for the control. CT18 (and ZT18 for cAMP assay) were selected as time of treatment due to significant circadian phase shifts observed on human melatonin phase response curves, and we hypothesized any cellular effects of melatonin would be most pronounced at this time [17,48]. Two hours after treatment, luminescence was recorded for each of the samples and used to calculate percent difference in FRQ levels from the reading immediately before interruption: (relative luminescence reading at 0 hr /relative luminescence reading at 2 hr) x100.

### Preparation of samples for cAMP-Glo^TM^ assay

To measure changes in cAMP concentration in response to melatonin, the Promega cAMP-Glo^TM^ Assay was used [49]. The assay is designed for mammalian cells but was successfully adapted for fungi in a previous study and works like a cell suspension in the assay protocol [50]. Methodology from previous literature was slightly altered in the present study for use with *N*. *crassa*. Cultures of N380-1, N380-2, and *gna-*1 were removed from LD12:12 conditions at ZT12. Under red light, a conidia suspension was made for each sample by adding 25mL of high glucose liquid medium (HGM, 2% D-glucose, 0.5% L-arginine, 1X Vogel’s salts) to the culture flasks and vortexing for 30 seconds. The suspension was filtered through a single layer of miracloth into centrifuge tubes. Cell counts were taken, and suspensions were diluted with HGM until all cell counts were roughly around 1.0x10^7^ cells/mL. In constant darkness and while nutating, the conidia suspensions were left to culture until ZT18 to normalize cell age. At ZT18, 10ml of the suspension was aliquoted into separate tubes and treated with 1ml of 5ng/mL melatonin solution or sterile water as a control and nutated for 10 minutes at room temperature in constant darkness. The samples were spun at 2000rpm, 4°C for 10 minutes. Pellets were dried and resuspended in 1.5mL of ice-cold sterile water and spun again for 7 minutes at the same settings. The pellets were dried again and 500mg of zirconia silica beads and 1mL ice-cold 10% TCA were added to the tubes. The samples were beat for 15 minutes in an ice-cold beater at max speed and then spun for 7 minutes at 3000rpm. The liquid layer was removed and washed in glass test tubes twice with water-saturated ether. Following the cAMP-Glo^TM^ assay protocol for recommended test compound volumes for cell suspensions in a 96-well plate, 10μL of the aqueous layer was used in each test well. Each strain and treatment group had 6 technical replicates. From this point forward, the cAMP-Glo^TM^ protocol was followed for the 96-well assay format.

## Supporting information

S1 Fig Decrease of cellular cAMP levels in response to a concentration range of melatonin treatments. Melatonin was dissolved in HGM and serially diluted. Control was treated with HGM containing no melatonin. **—p<0.005, ***—p<0.0005.
(TIFF)

S2 Fig Dali structural alignment of *N*. *crassa* GPR-3 to MT1(left) and MT2 (right). Uppercase letters denote structurally equivalent positions, vertical lines denote equivalent amino acid positions. Query–GPR-3, Subject–MT1 or MT2, DSSP abbreviations–H(helix), L(loop), E(coil). Z-score = 21.1, 20.5, respectively.
(TIFF)

S1 Data Raw data for Figs 3, 4 and S1. (XLSX)
