# Supplementary figures and images for: Cross-species comparison of AlphaFold-derived G protein-coupled receptor structures reveals novel melatonin-related receptor in Neurospora crassa
Source: PLoS One. 2025 Jan 28;20(1):e0318362. doi: 10.1371/journal.pone.0318362 (PMC11774363; doi:10.1371/journal.pone.0318362)

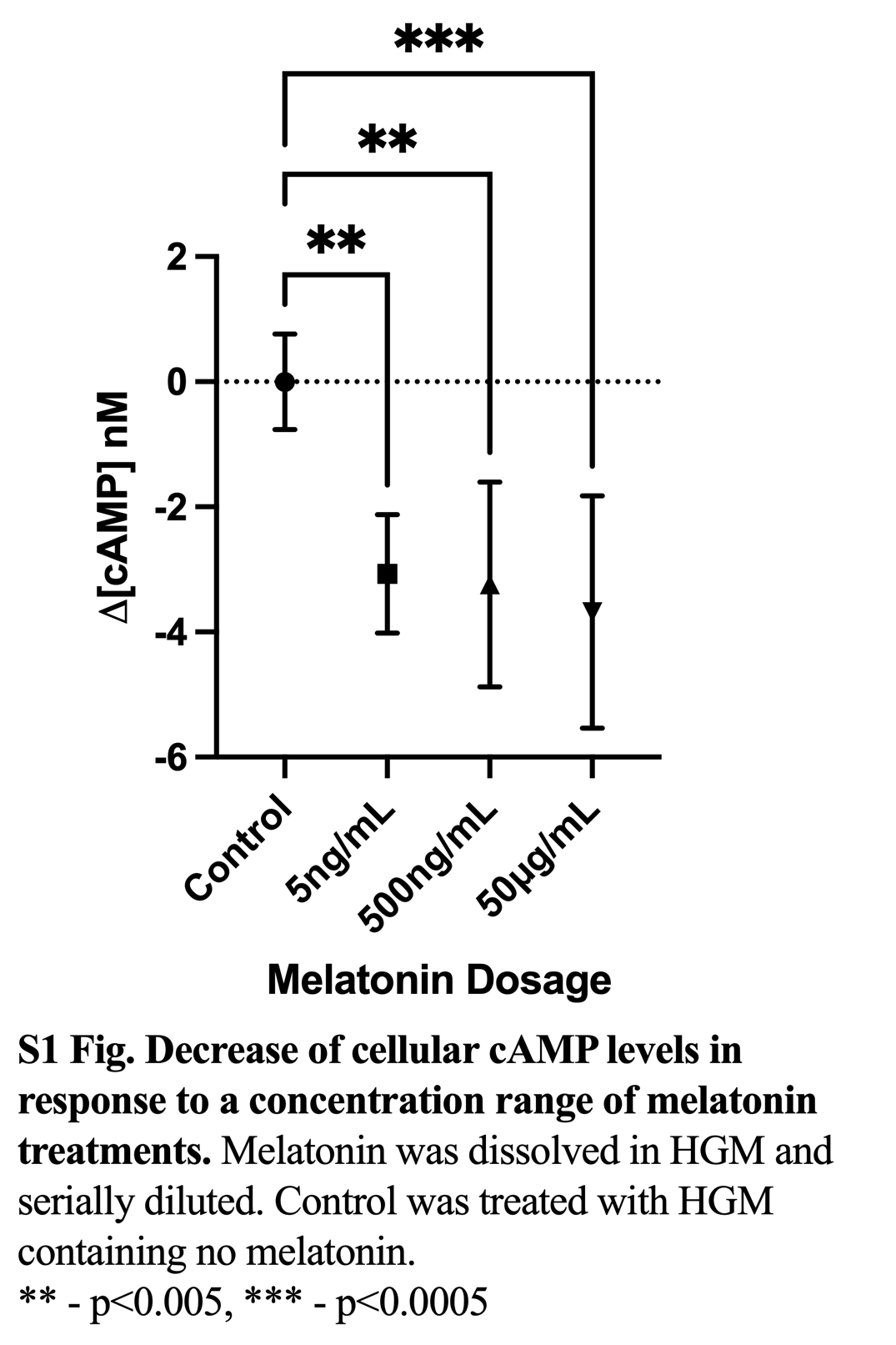

Supplement: S1 Fig — Melatonin was dissolved in HGM and serially diluted. Control was treated with HGM containing no melatonin. **—p<0.005, ***—p<0.0005. (TIFF) [file pone.0318362.s001.tiff]

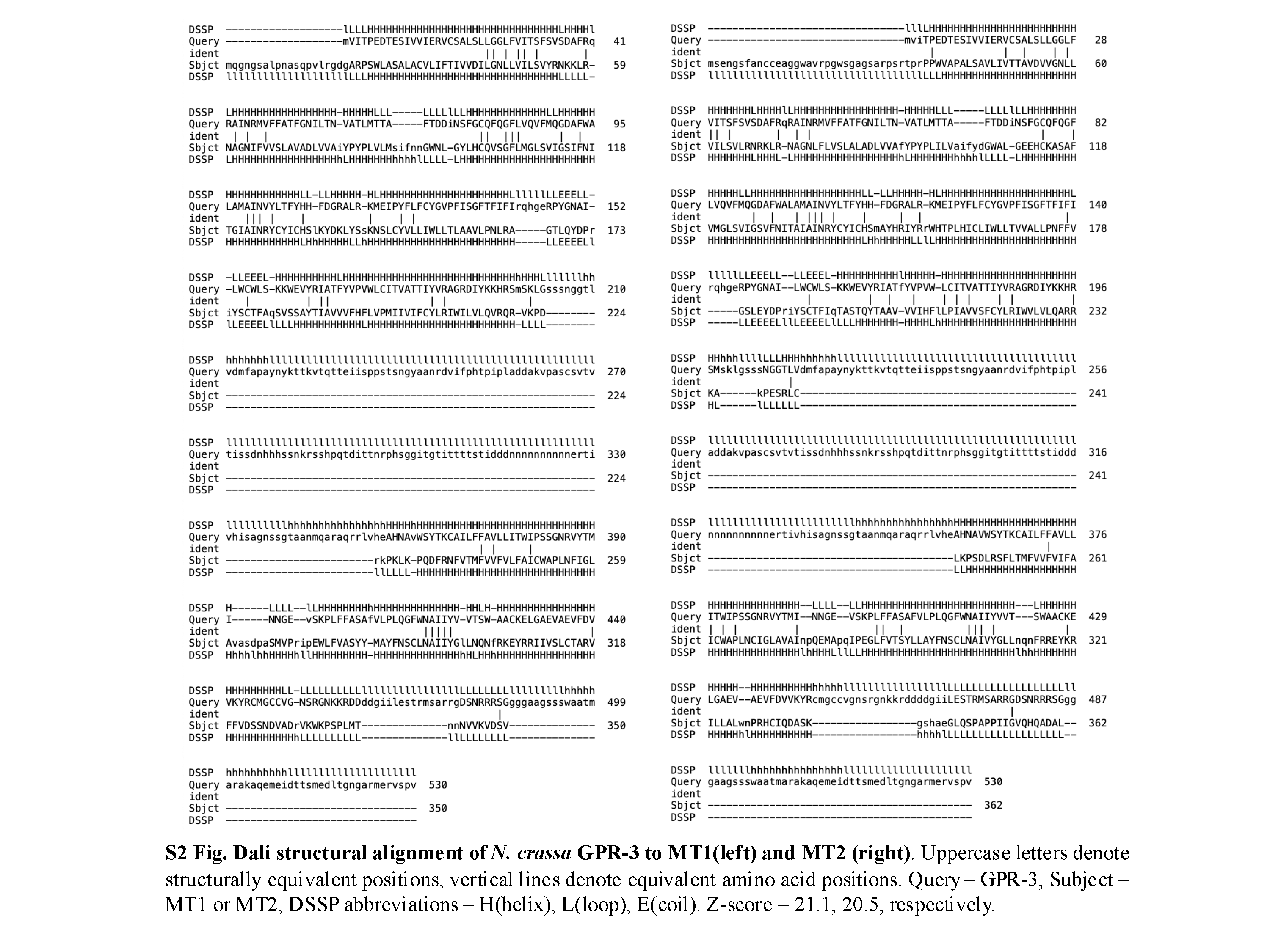

Supplement: S2 Fig — Dali structural alignment of N. crassa GPR-3 to MT1(left) and MT2 (right). Uppercase letters denote structurally equivalent positions, vertical lines denote equivalent amino acid positions. Query–GPR-3, Subject–MT1 or MT2, DSSP abbreviations–H(helix), L(loop), E(coil). Z-score = 21.1, 20.5, respectively. (TIFF) [file pone.0318362.s002.tiff]
